# The Management of Infected Oroantral Fistula After Maxillary Third Molar Removal: A Case Report

**DOI:** 10.7759/cureus.42633

**Published:** 2023-07-28

**Authors:** Chinnaiah R, Sujith Raj Stephen, Veeramuthu M, Satheesh G, Rajashri R

**Affiliations:** 1 Department of Oral and Maxillofacial Surgery, Adhiparasakthi Dental College and Hospital, Melmaruvathoor, IND; 2 Department of Dentistry, Panimalar Medical College Hospital and Research Institute, Chennai, IND; 3 Department of Oral and Maxillofacial Surgery, Karpaga Vinayaka Institute of Dental Sciences, Chengelpet, IND

**Keywords:** nasal regurgitation, wisdom tooth extraction, maxillary sinusitis, oroantral communication, oroantral fistula

## Abstract

Oroantral communication is an unnatural communication of the maxillary sinus with the oral cavity, often resulting from dental extractions, infection, trauma, or excision of cysts or tumors. Pathological epithelialization of oroantral communication leads to oroantral fistula. Various techniques have been proposed for surgical closure. Uneventful healing of the defect can be achieved in the absence of antral infection. Hence, medical management of maxillary sinusitis should precede surgical closure of the defect. Here, we report a case of an oroantral fistula of the left maxillary third molar, caused by a secondary infection of the extraction site, managed primarily by antibiotics, topical steroids, and irrigating agents followed by surgical closure. It is essential to carefully inspect the post-extraction socket of maxillary teeth due to its high risk of development of oroantral communication. Also, the management of oroantral communications needs early detection to prevent infection and to prevent transforming into an oroantral fistula. In case of an infected oroantral communication or fistula, priority rests on treating the infection first and followed by surgical repair.

## Introduction

Oroantral communicating defects can cause an infection of the maxillary antrum because it acts as a pathological pathway for bacteria from the oral cavity to the maxillary sinus cavity. Oroantral communicating defect occurs following procedures like tooth extraction with or without ostectomy, resection of malignant tumors, or removal of cysts and benign tumors, which obstructs the healing process. If the oroantral communication (OAC) remains open, it becomes epithelialized and develops into an oroantral fistula (OAF). Extraction of maxillary molars with long roots and in close approximation with a large antrum is the most common cause of OAC [1]. OAC may also result from the extraction of maxillary third molars, particularly when surgery is required, either with or without ostectomy [2]. Once OAC or OAF has been diagnosed, surgical closure is mandatory. Surgical closure of the OAC/OAF can be done using rotating or advancing soft tissues such as the buccal flap, palatal flap, submucosal tissue, buccal fat pad, and tongue flap [1]. However, successful closure of chronic OAF can be achieved if there is an absence of infection in the maxillary sinus. Therefore, the first aim is to eliminate the infection from the maxillary sinus before treating the OAF [3]. This report presents a case of OAF following the surgical removal of an impacted left maxillary third molar. Maxillary sinusitis had been diagnosed postoperatively and treated medically prior to the successful surgical closure of the oroantral communicating defect.

## Case presentation

**Figure 1:**
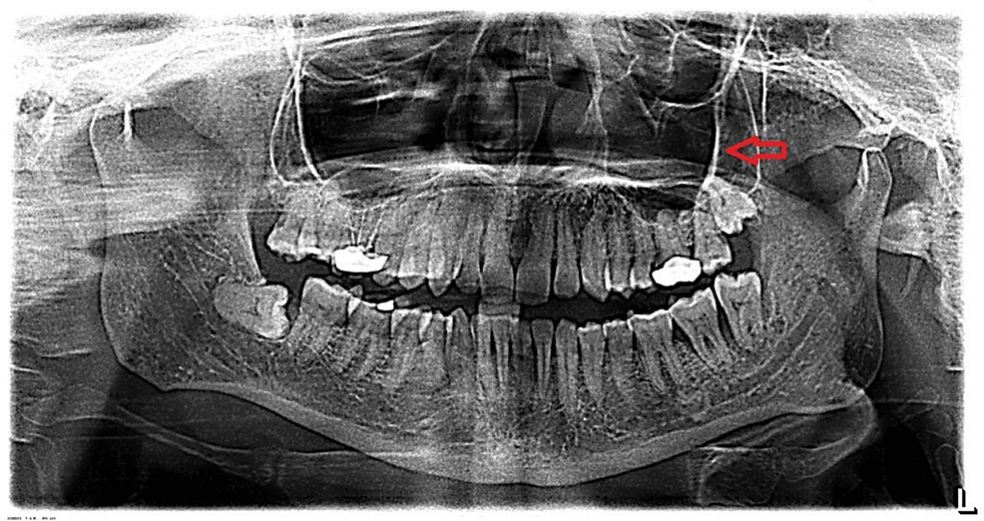
Panoramic x-ray shows impacted left maxillary third molar which is closely related to the maxillary sinus

A 50-year-old male patient presented with a chief complaint of pain in the left upper and lower back teeth region. Panoramic x-ray showed impacted left maxillary third molar, closely related to the maxillary sinus (Figure 1).

**Figure 2:**
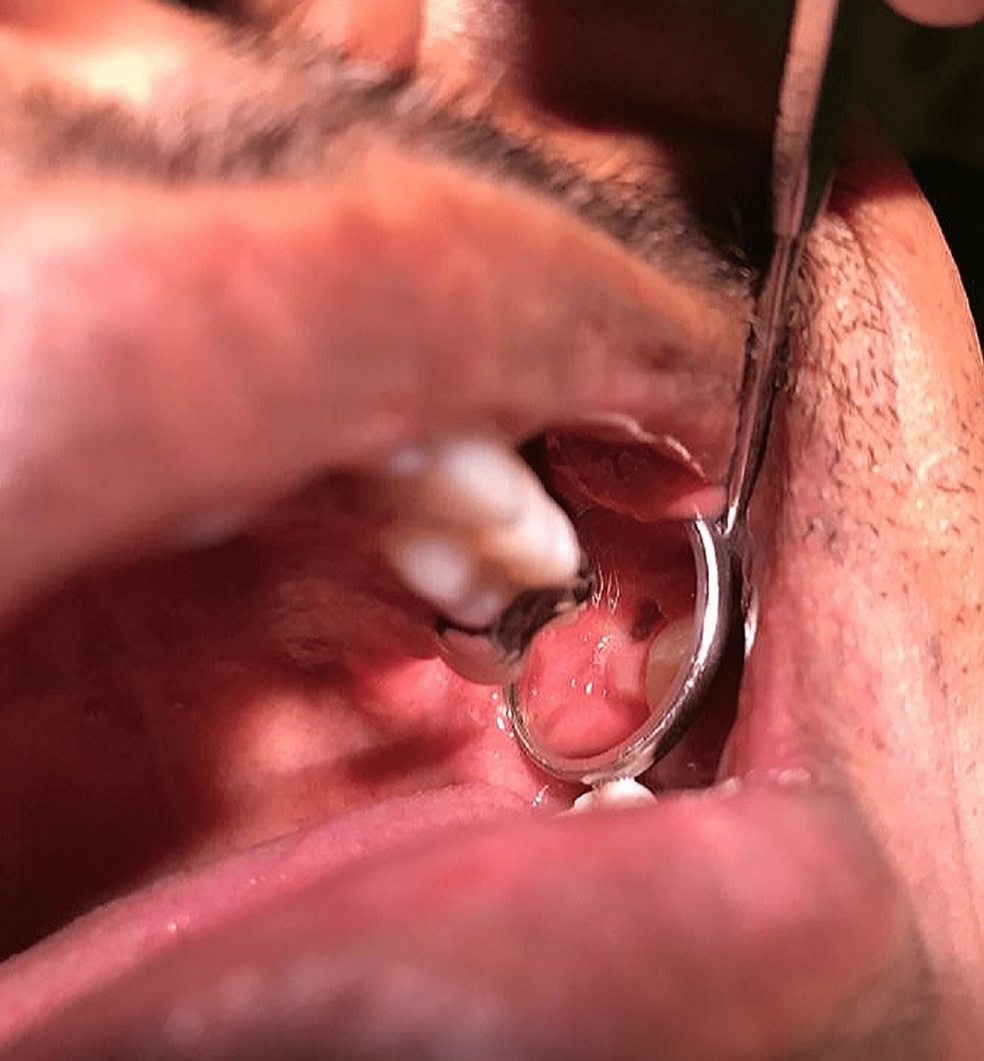
Oroantral fistula seen intraorally

Under local anesthesia, an incision was placed, the mucoperiosteal flap was elevated, bone guttering was done, and the tooth was luxated and extracted. Primary closure of wound done with simple interrupted sutures. The patient was specifically advised to avoid nose blowing and forceful gargling for one week and was prescribed a course of amoxicillin and clavulanic acid 625 mg twice daily for five days along with routine postoperative analgesics and instructions after extraction. After the surgery, the patient did not follow up with review appointments on the third and the fifth postoperative days but reported with a complaint of fluid leakage while drinking and gargling of water through his left nostril on postoperative day 10. He also complained of pain in the left side of the face with a foul smell. On examining intraorally, it was seen that the extraction socket did not heal satisfactorily and an opening of 5 mm diameter was present. The socket was filled inadequately with granulation tissue (Figure 2). 

When the socket was irrigated with normal saline, minimal fluid leakage was present in the left maxillary sinus. A diagnosis of OAF was made and the patient was prescribed oral antibiotics to reduce bacterial load, antihistamine to reduce allergic rhinitis, mucolytic agent for making the mucus thin to ease drainage, nasal decongestant to reduce acute nasal congestion, steroid spray to reduce inflammation, and 3% sodium chloride drops to improve mucociliary clearance and decongesting the nose through an osmotic mechanism (Table 1).

**Table 1:** Drug regimen HS: *hora somni* (bed time); BD: *bis in die* (twice daily); NACL: sodium chloride; Tab: tablet

Drugs	Dosage	Frequency	Days
Tab levofloxacin (antibiotic)	500 mg	BD	5 days
Tab levocetirizine (antihistamine)	10 mg	HS	5 days
Tab ambroxol (mucolytic agent)	30 mg	BD	5 days
Oxymetazoline nasal drops (nasal decongestant)	2 drops	BD	2 days
Fluticasone nasal spray (steroid)	2 puffs	BD	3 days
Hypertonic saline drops, 3% NACL (decongestant)	3 - 4 drops	BD	3 days

**Figure 3:**
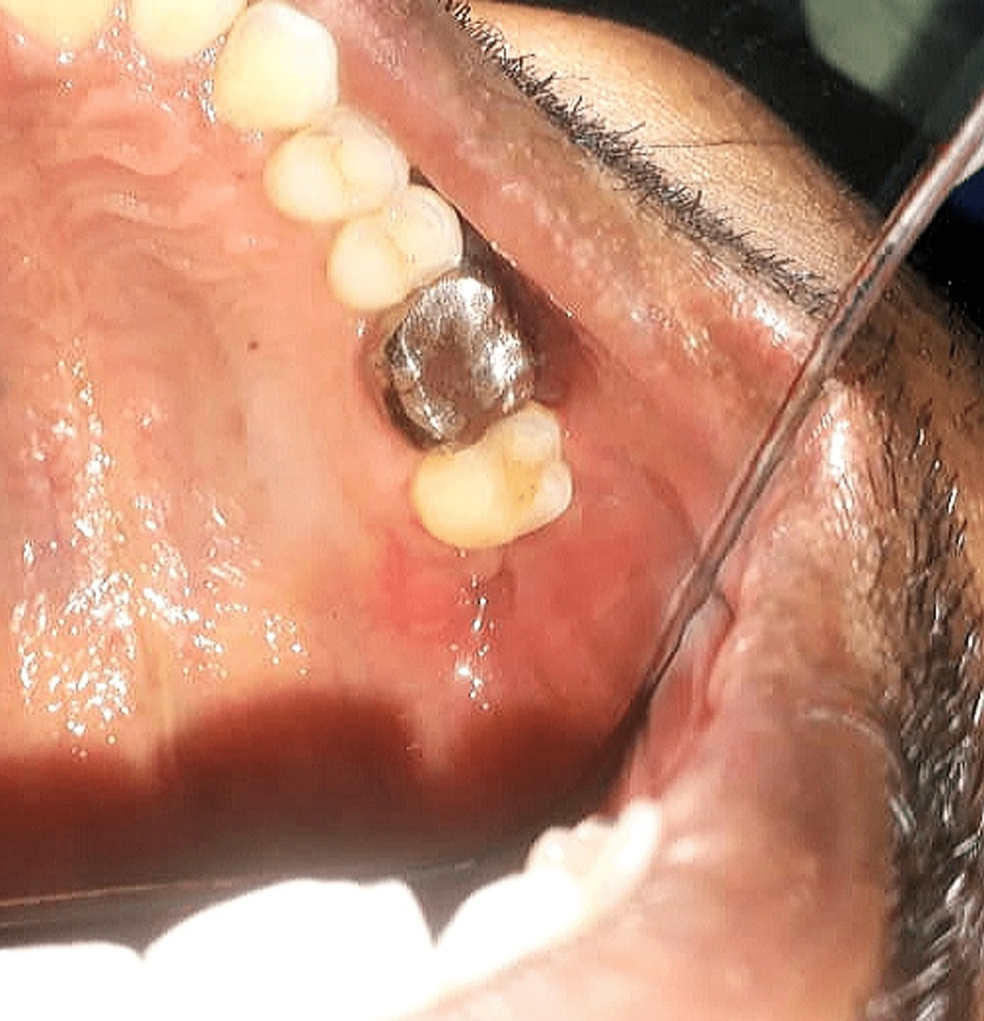
Satisfactorily healed wound after primary closure

Five days later, the patient was operated on for the closure of the OAF with crestal incision and an anterior relieving incision, a full-thickness mucoperiosteal flap was raised, the fistula tract was excised, and the buccal flap advanced. Vertical mattress sutures were placed and postsurgical instructions were given. One week after the surgery, the wound healed satisfactorily with cessation of fluid leak from mouth to his left nostril (Figure 3). 

## Discussion

The most prevalent cause of OAC is the surgical removal of the second premolar and first and second molars of the upper jaw, due to the close approximation of the root apex to the maxillary sinus [4]. During the third decade of life, the maxillary sinus reaches its maximum size, due to which the incidence of OAC increases beyond the age of 30 [5]. After the extraction of upper posterior teeth, the incidence of OAC followed by subsequent transformation into OAF ranges between 0.31% to 5.1% [6].

The complication can occur in the case of maxillary third molar extractions, particularly when an aggressive surgical technique is employed or when the patient performs maneuvers immediately after surgery which increases the intra-antral pressure [2]. In the present case, the close relationship of the left maxillary third molar to the inferior antral wall predisposes a higher chance of incidence of OAC. The risk of such communication became higher when surgical removal of overlying bone in the impacted maxillary third molar was performed. Valsalva maneuver is a clinical test to diagnose OAC/OAF. The test is performed by closing both the nostrils and keeping the mouth open and blowing through the nose gently. In the presence of OAC/OAF, a whistling sound is heard as the air passes down the fistula into the oral cavity. It can also be observed as bubbles of air, mucous secretion, or blood around the opening. The use of a blunt-edged Bowman probe to examine perforations in the floor of the maxillary sinus can be used to detect OAC intraoperatively [7,8]. The operator may fail to notice the occurrence of such communication because it is not in standard practice to check after every tooth extraction; therefore, no specific treatment is done to close the defect. In the present case, OAC was not suspected after the removal of the left maxillary third molar; thus, no specific measure was performed to close it.

When the oroantral defect measures less than 2 mm in diameter, it usually closes spontaneously, but when the defect is larger than 3 mm, or in the presence of periodontal or antrum inflammation, the defect often persists and progresses into chronic maxillary sinusitis. Sinusitis is characterized by the presence of the following symptoms like pain, headache, nasal obstruction, purulent nasal secretion, and postnasal drip. If the OAC is not treated, there is an invasion of microorganisms from the oral cavity which exacerbates the infection of the antrum and may cause permanent epithelization of the communication leading to OAF, further increasing the risk of sinusitis [4,9].

According to Howe, the only way to achieve a successful closure of chronic oroantral fistula is when there is an absence of antral infection [10]. Therefore, the first goal is to treat any coexisting maxillary sinus infection. Likewise, the defect in the present case was closed by surgical intervention following conservative treatment using a combination of oral antibiotics, topical nasal steroids, and hypertonic antral irrigation [11,12].

## Conclusions

Two important things are highlighted in this case study. First, it is recommended to thoroughly examine the socket after removing the impacted upper third molar, which is closely approximated with the maxillary sinus, and to see whether OAC exists. Second, regardless of the surgical technique, it is important to treat any pre-existing antral infection before planning the primary closure of OAF.
